# A Chemoinformatics Investigation of Spectral and Quantum Chemistry Patterns for Discovering New Drug Leads from Natural Products Targeting the PD-1/PD-L1 Immune Checkpoint, with a Particular Focus on Naturally Occurring Marine Products

**DOI:** 10.3390/md23060247

**Published:** 2025-06-10

**Authors:** Henrique Rabelo, Ayana Tsimiante, Yuri Binev, Florbela Pereira

**Affiliations:** 1 LAQV-REQUIMTE, Department of Chemistry, NOVA School of Science and Technology, Universidade Nova de Lisboa, 2829-516 Caparica, Portugal; h.rabelo@campus.fct.unl.pt (H.R.); ayana.tsimiante@etu.sorbonne-universite.fr (A.T.);; 2 Faculté des Sciences & Ingénierie, Sorbonne Université, 75006 Paris, France

**Keywords:** natural products (NPs), immuno-oncology, PD-1/PD-L1 immune checkpoint, machine learning (ML) techniques, molecular docking, nuclear magnetic resonance (NMR), quantitative structure–activity relationship (QSAR) models, virtual screening

## Abstract

(1) Background: Although the field of natural product (NP) drug discovery has been extensively developed, there are still several bottlenecks hindering the development of drugs from NPs. The PD-1/PD-L1 immune checkpoint axis plays a crucial role in immune response regulation. Therefore, drugs targeting this axis can disrupt the interaction and enable immune cells to continue setting up a response against the cancer cells. (2) Methods: We have explored the immuno-oncological activity of NPs targeting the PD-1/PD-L1 immune checkpoint by estimating the half maximal inhibitory concentration (IC_50_) through molecular docking scores and predicting it using machine learning (ML) models. The LightGBM (Light Gradient-Boosted Machine), a tree-based ML technique, emerged as the most effective approach and was used for building the quantitative structure–activity relationship (QSAR) classification model. (3) Conclusions: The model incorporating 570 spectral descriptors from NMR SPINUS was selected for the optimization process, and this approach yielded results for the external test set with a sensitivity of 0.74, specificity of 0.81, overall predictive accuracy of 0.78, and Matthews correlation coefficient (MCC) of 0.55. The strategy used here for estimating the IC_50_ from docking scores and predicting it through ML models appears to be a promising approach for pure compounds. Nevertheless, further optimization is indicated, particularly through the simulation of the spectra of mixtures by combining the spectra of individual compounds.

## 1. Introduction

The statistical analysis of new drug approvals by the Food and Drug Administration (FDA) from 2011 to 2020 reveals a significant upward trend in the annual approval rate of new molecular entities (NMEs) [1]. During this period, the 10-year average reached 31.4 NMEs per year, a notable increase compared to the previous decade (2001–2010), which averaged 18.4 NMEs annually [1,2,3,4]. In contrast, natural products (NPs) and NP derivatives showed steady approval rates over time, with a 10-year average of 4.2 and 3.8 drugs per year for the decades 2001–2010 and 2011–2020, respectively [1,2,3,4]. Interestingly, the peak approval for NPs and NP derivatives occurred in 1996, with 12 drugs approved, and the 1990s were also the most prolific for computer-aided drug design (CADD)-driven drugs, with eight approvals [2]. Additionally, over half of all approvals for marine natural products (MNPs) and MNP derivatives were in the 21st century, with eight of the eleven approved drugs occurring after 2000 [1,2,3,4]. New strategies are essential to address the perceived disadvantages of NPs compared to synthetic drugs, including challenges in accessibility and supply that delayed MNP research until the 1980s [2,5,6]. For example, the development of marine-derived drugs is a lengthy and costly process, taking between 17 years (e.g., trabectedin) and 24 years (e.g., halichondrin; dolastatin), with an average of 23 years from the initial discovery of a MNP to its market approval [5]. To mitigate these challenges, CADD approaches can support decisions on the in vivo and in vitro testing of isolated NPs and extracts, facilitate the design of bioactive NP derivatives, and enable virtual screening of databases containing known or proposed NPs. The COlleCtion of Open NatUral producTs (COCONUT), an open-access database of NPs, launched in 2021 at https://coconut.naturalproducts.net (accessed on 3 March 2025) [7], is one of the largest resources available for NP annotation [7,8]. The database contains structures of over 695,000 unique NPs, including 82,220 molecules without stereocenters, 539,350 molecules with defined stereochemistry, and 73,563 molecules with stereocenters but undefined absolute stereochemistry [8]. The complete set of structures is available for download in SDF, CSV, and database dump formats, enabling integration with other structural feature-based databases for dereplication purposes [8].

Understanding where biologically relevant compounds reside within the chemical structural space—and how these two realms, chemical and biological, intersect—is crucial for unlocking new insights and driving innovation in the exploration of NPs. The regions of chemical space surrounding NPs are widely recognized as highly promising for the development of new drug leads, as demonstrated in a comprehensive analysis spanning the period from 1981 to September 2019. NP scaffolds—including unaltered NPs, NP derivatives, NP mimetics, and structures containing an NP pharmacophore—account for 45% of all approved small-molecule drugs [9]. A structural classification analysis of NPs conducted by Waldmann and colleagues [10] revealed that over half of all NPs possess an optimal size (a van der Waals volume between 300 and 800 Å^3^), making them well-suited as starting points for hit-to-lead discovery. Similarly, Pereira [11] reported a strong correlation between active compounds and three- or four-ring structures with a van der Waals volume within the same range in a distinct PubChem subset. To further enhance the exploration of NP chemical space, Ertl et al. [12] developed an NP-likeness score, which quantifies the similarity of a molecule to the structural features of NPs. This metric has been integrated into the COCONUT database, providing researchers with a valuable tool for drug discovery and development [8]. Recently, two complementary studies have shed light on the distinctions between terrestrial natural products (TNPs) and marine natural products (MNPs) [11,13]. Shang et al. [13] utilized chemoinformatics methods to analyze these differences, while Pereira [11] applied ML modeling to predict the terrestrial or marine origin of NPs. Both studies highlighted a trend where MNPs tend to contain more halogens (particularly bromine) and fewer oxygen-containing groups compared to TNPs [11,13]. However, the studies reached different conclusions regarding the ring size. Shang et al. [13] reported that larger rings, particularly those with 8 to 10 members, were more prevalent in MNPs. In contrast, Pereira [11] found that five-membered rings were more significant in distinguishing MNPs. Despite these discrepancies, Pereira [11] observed a clear separation between the chemical spaces occupied by MNPs and TNPs when employing ML techniques, underscoring the distinct nature of these two groups of NPs.

The clinical translation of immune checkpoint inhibitors (ICIs), which modulate T-cell activation, was the most significant advancement in cancer treatment over the past decade [14]. Currently, eight PD-1/PD-L1 ICIs have been approved [14]. While all approved ICIs are monoclonal antibodies (mAbs), they present challenges such as poor oral bioavailability, prolonged tissue retention, low membrane permeability, and high costs. As a result, research has shifted toward developing small-molecule inhibitors to address these limitations [14]. Few studies have explored CADD for PD-1/PD-L1 inhibition [15], with most relying on Structure–Activity Relationship (SAR) analysis and PD-L1 docking, mainly based on Bristol–Myers Squibb (BMS) compounds [16,17,18,19,20,21]. Our group recently reported an integrated CADD approach combining QSAR modeling, drug repurposing, and molecular docking, offering a novel strategy for PD-1/PD-L1 inhibition [22]. In the same study, sonidegib, an anticancer drug with a biphenyl system, was identified as a potential hit and later validated for in vitro PD-1/PD-L1 binding modulation using ELISA and flow cytometry [22].

The ability to predict biological activity through inexpensive, rapid, and automated testing of extracts is a transformative technology that enables the selection of promising extracts prior to isolation and structure elucidation of compounds. Biological activity is a consequence of molecular structure, and the latter is reflected in NMR spectra. For several classes of compounds, ML techniques have been used to automatically extract structural features [23] and also to predict physical/chemical properties [24] and biological activities [25] from NMR spectra—Quantitative Spectrometric Data–Activity Relationships (QSDAR)—and ^13^C NMR spectra have been found to be necessary in addition to ^1^H NMR. Although their predictive power is generally lower than that of QSAR, QSDAR has the enormous advantage of not requiring the structure of the compound (only its spectra). Latino & Aires-Sousa showed that ML algorithms can classify photochemical and metabolic reactions from the difference between the ^1^H NMR spectra of the products and the reactants [26,27]. Recently, the use of QSDAR models to discover new inhibitors against the human colon carcinoma HCT116 cell line [28] and methicillin-resistant *Staphylococcus aureus* (MRSA) infection [29] has also been reported by Pereira and co-workers. The QSDAR classification models were built using the experimental ^1^H and ^13^C NMR spectra obtained from 50 crude extracts, 55 fractions, and 50 pure compounds from actinobacteria isolated from marine sediments collected off the Madeira Archipelago [28,29].

The present study was undertaken to investigate the immuno-oncological activity of NPs that target the PD-1/PD-L1 immune checkpoint. To this end, the IC_50_ was estimated on the basis of molecular docking scores and predicted using ML models. The dataset was extracted from the COCONUT database and comprised 120,935 molecules with a HeavyAtomMolWt (the average molecular weight of the molecule, ignoring the hydrogens) of less than 500 Da. All 120,935 NPs in the dataset were subjected to molecular docking against the PD-L1 receptor (PDB ID 5N2F) in order to predict the conformations and scores associated with the docking process. The QSDAR models for pure compounds were developed using two distinct approaches to spectral data calculation. The first approach employed a graph neural network (GNN) model to predict NMR chemical shifts—NMR GNN. The second approach utilized the SPINUS program (https://neural.dq.fct.unl.pt/spinus/, (accessed on 3 March 2025)) to predict both ^1^H NMR chemical shifts and coupling constants—NMR SPINUS. The support vector machine (SVM), convolutional neural network (CNN), RF, and light gradient-boosted machine (LightGBM) were the four machine learning techniques explored. The objective was to predict PD-1/PD-L1 inhibition, with the performance of the models assessed through internal and external validation. The tree-based ML technique, LightGBM, was identified as the most effective approach and was therefore employed for the construction of the QSDAR classification model. The model comprising 570 spectral descriptors derived from SPINUS exhibited the most favorable performance for the test set, with a sensitivity of 0.74, a specificity of 0.81, an overall predictive accuracy of 0.78, and an MCC of 0.55. Furthermore, the performance of these QSDAR models was benchmarked against that of conventional QSAR models utilizing molecular, fingerprint, and quantum descriptors. The strategy of estimating the half maximal inhibitory concentration (IC_50_) from docking scores and predicting it through ML models appears to be a promising approach for pure compounds. Nevertheless, further optimization is possible, particularly through the simulation of mixtures by combining the spectra of individual compounds.

## 2. Results and Discussion

### 2.1. Docking Score for the Estimation of IC_50_ Values

**Figure 1:**
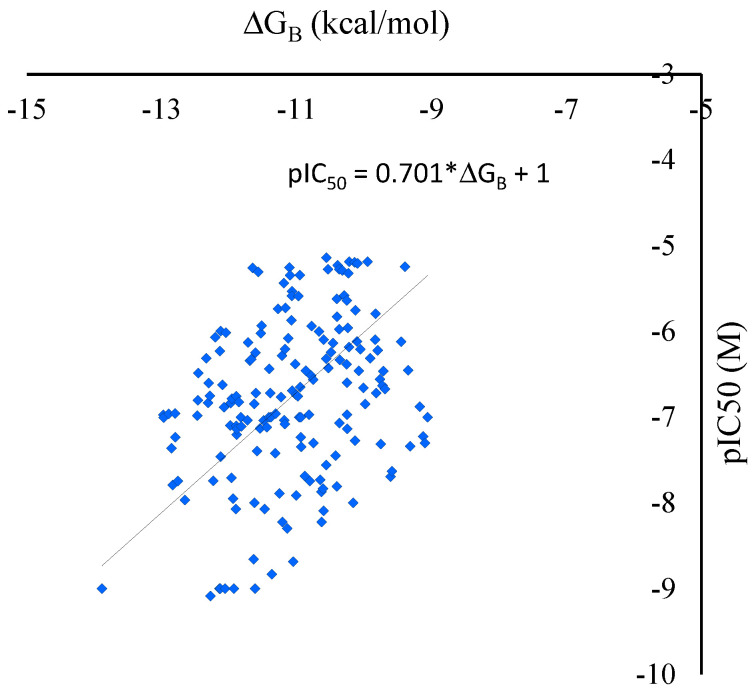
Experimental pIC_50_ *versus* ΔG_B_, calculated against PD-L1 for 172 active molecules.

The potential of utilizing the calculated free binding energies (ΔG_B_) by molecular docking with AutoDock Vina (version 1.2.3) was investigated as an output of the QSDAR model. A total of 172 molecules with a HeavyAtomMolWt of less than 500 Da, as reported in the literature as active against PD-1/PD-L1, were subjected to molecular docking with PD-L1. The correlation between pIC_50_ and ΔG_B_ for these molecules was subsequently analyzed (Figure 1).

**Figure 2:**
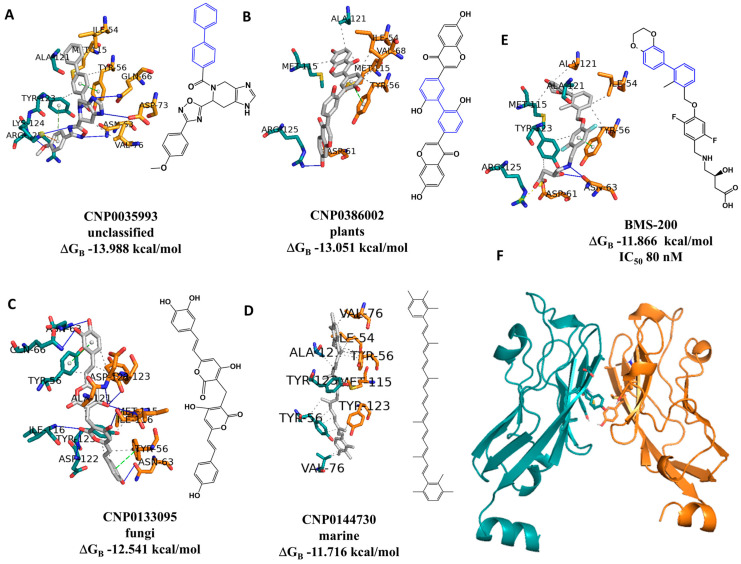
Interaction profiles of the best-docked poses for the (**A**) CNP0035993 (unclassified), (**B**) CNP0386002 (plants), (**C**) CNP0133095 (fungi), (**D**) CNP0144730 (marine), and (**E**) BMS-200 (positive control) against PD-L1 (PDB ID: 5N2F) with the calculated ΔG_B_ of −13.988 kcal/mol, −13.051 kcal/mol, −12.541 kcal/mol, −11.716 kcal/mol and −11.866 kcal/mol, respectively. (**F**) The co-crystal dimer structure of PD-L1 (PDB ID: 5N2F) with chains A and B is highlighted in cyan and orange, respectively. BMS-202 also has an IC_50_ determined by the homogeneous time-resolved fluorescence (HTRF) binding assay [30].

As can be seen in Figure 1, there is a correlation between the docking scores and the experimental pIC50 values (R = 0.32). Furthermore, we have complete control over the calculated values, i.e., all molecules are calculated using the same procedure. This is something we cannot guarantee when using experimental values from different sources. Therefore, all 120,935 NPs in the dataset were docked to the PD-L1 receptor (PDB ID 5N2F) to predict docking conformations and scores. The interactions of the best-docked unclassified NP, CNP0035993, plant NP, CNP0386002, fungal NP, CNP0133095, and MNP, CNP0144730, with PD-L1 were analyzed and compared to the positive control, BMS-200 (Figure 2).

Figure 2 illustrates the chemical structure of one of the predicted NPs with the lowest ΔG_B_ (**A**). The structure features the biphenyl moiety, highlighted in blue, along with the predicted binding pose. It can be observed that there are different binding poses for the two NPs above (**C** and **D**) compared to the positive control (BMS-200, **E**), and in particular, the interaction with Tyr56 of chain A. The two NPs from the fungal and marine sources depicted in the images (**A** and B) were found to exhibit binding poses that were similar to those of the positive control. The experimental IC_50_ value displayed in Figure 2, 80 nM, for BMS-200 (**E**) can be compared with the value of IC_50_ estimated by molecular docking, which was found to be 48.08 nM.

The SMILES strings of the 172 molecules comprising the active dataset (as reported in the literature as being active against PD-1/PD-L1) are available as Supplementary Material, File S1, along with the corresponding experimental activities (IC_50_) and estimated ΔG_B_.

### 2.2. QSDAR Regression Modeling

#### 2.2.1. Dataset

The dataset was extracted from the COCONUT database and consists of more than 120,000 molecules with a HeavyAtomMolWt of less than 500 Da, which were subjected to molecular docking against the PD-L1 protein and spectral data calculations.

**Figure 3:**
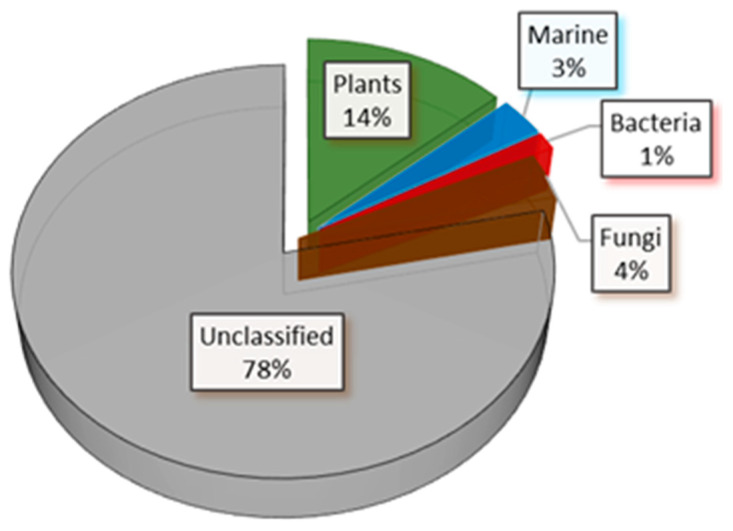
The source origin distribution of the dataset extracted from the COCONUT database.

The dataset displays the following distribution: the majority remains unclassified, with 14% originating from plant-derived natural products, 3% from marine sources, 1% from bacteria, and 4% from fungi; Figure 3.

The entire dataset, comprising 120,935 NPs, was randomly partitioned into a training set of 119,733 molecules and a test set of 1202 molecules.

These sets were employed for the development (training set) and external validation (test set) of the QSDAR regression models. It is generally accepted that a given compound exhibits considerable inhibitory activity when the IC_50_ is less than or equal to 10 µM. This value is typically employed to differentiate between active and inactive compounds. Table 1 presents an analysis of the dataset, which has been divided into training and test sets. These have been fractionated by active and inactive categories, and the drug-likeness characteristics (e.g., HeavyAtomMolWt, MolLogP) and the NP-likeness score have also been considered.

As evidenced by the analysis in Table 1, the test set appears to be a reliable representation of the training set. This is evident from the comparable distribution of active and inactive NPs between the two sets, as well as from the consistency in drug-likeness and NP-likeness parameters. A significant proportion of NPs, exceeding 84%, in both the training set and the test set adhere to two of Ghose’s rules [31], thereby substantiating the high degree of drug-likeness inherent to the dataset derived from the COCONUT database. Ertl et al. [12] developed the NP-likeness score and mapped this score onto the various chemical spaces. It was observed that for an NP-likeness between −1 and 1, there seems to be a higher percentage of drugs. Thus, for our dataset, the percentage of NPs with the highest similarity with drugs is greater than or equal to 43%.

#### 2.2.2. Spectral Data

*NMR GNN*: The NMR chemical shifts were predicted by a graph neural network (GNN) model developed by Yang et al. in Python, version 1.1.0 [32]. The chemical shifts and types (H, C, N, O, etc.) were encoded by a fixed-length numerical code of 250 spectral descriptors.

*^1^H NMR SPINUS* (Structure-based Predictions In NUclear magnetic resonance Spectroscopy): ^1^H NMR chemical shifts and coupling constants were predicted by ensembles of feed-forward neural networks (FFNN) and were incorporated into Associative Neural Networks (ASNN), https://neural.dq.fct.unl.pt/spinus/, (accessed on 3 March 2025) [33]. A fixed-length numerical code of 570 spectral descriptors encoded the discrete values of the ^1^H chemical shifts and coupling constants.

#### 2.2.3. QSDAR Model Development

The RF ML technique was employed for the construction of QSDAR models to predict PD-1/PD-L1 inhibition based on two distinct NMR spectral descriptors (e.g., SPINUS, GNN). The efficacy of the models was successfully evaluated through internal validation (OOB estimation for the training set), as illustrated in Table 2.

The model comprising 820 spectral descriptors derived from NMR GNN and SPINUS exhibited the most favorable performance for the training set, with a MAE of 0.23, RMSE of 0.30, and an R^2^ of 0.91. Although the QSDAR model with the NMR SPINUS descriptors shows a slightly reduced predictive capacity compared to the NMR SPINUS and GNN models for the training set, with a MAE of 0.23, a RMSE of 0.31, and an R^2^ of 0.91, it is selected for the subsequent optimization process. This is primarily due to its capacity to more accurately reproduce the experimental NMR spectrum whilst also utilizing the prediction of ^1^H NMR data. In different experiments, the prediction of ^1^H NMR chemical shifts and coupling constants with SPINUS achieved mean absolute errors (MAE) of 0.16–0.35 ppm and 0.6–0.8 Hz, respectively [33]. A reference NMR prediction tool, NMRShiftDB, has reported a similar MAE of 0.15–0.25 ppm [34]. Subsequently, this model was further optimized through descriptor selection based on the importance assigned by the RF model using the 50, 100, 150, or 200 most important descriptors, as illustrated in Table 3.

The selection of the 200 most important descriptors from the NMR SPINUS spectral descriptor set, used to build the model with the RF, enabled the training of much smaller RF models with equivalent prediction accuracies (R^2^ = 0.91 and MAE = 0.23) to those achieved by models trained with the complete set of descriptors for the training set. A comparison of four ML techniques using RF, LightGBM, SVM, and CNN for building the PD-L1 models with the 200 most important spectral descriptors selected by the RF descriptor importance is shown in Table 4.

It is clear that the best QSDAR model was achieved using the LightGBM algorithm. This is evidenced by the lower MAE and RMSE obtained for the training set in cross-validation estimation (see Table 4). However, the predictive capacity of the LightGBM model for the external set, the test set, decreases significantly, with a MAE for the test set that is considerably more than 2.82 times that obtained for the training set in cross-validation (see Table 4).

### 2.3. Benchmarking with QSAR Using Molecular, Fingerprint, and Quantum Descriptors

The performance of the QSDAR models constructed using spectral data that were reported in Section 2.2 was evaluated in comparison with more conventional QSAR models using fingerprints (FPs), 2D and 3D molecular descriptors, encompassing three distinct types of FPs with varying sizes (166 MACCS; 1024 Morgan, circular fingerprints and 2048 RDKit). A total of 242 1D&2D molecular descriptors were employed, encompassing electronic, topological, and constitutional descriptors, as well as three types of 3D molecular descriptors: Autocorr3D (80 descriptors), Getaway (271 descriptors), and Radial Distribution Function, RDF (210 descriptors). The RDKit software, version 2022.09.1, which is written in both C++ and Python, was employed for the calculation of molecular descriptors and FPs [35]. Fast estimation of the density functional theory (DFT) properties, previously developed by ML techniques in our group for molecular orbital energies [36] and dipole moment [37], enabled us to include five quantum descriptors, such as the energy of the highest occupied molecular orbital (εHOMO), the lowest unoccupied molecular orbital (εLUMO), the HOMO−LUMO gap, the dipole moment (DM) calculated using empirical point natural bond orbital (NBO) charges [38], and DFT-DM. The tree-based ML technique, LightGBM, was identified as the most effective approach in the QSDAR regression models and was also used to build the QSAR models. The models’ performance was successfully evaluated through internal validation (10-fold cross-validation for the training set), as depicted in Table 5.

The performance of the 3D descriptors in modeling activity against PD-L1 was noteworthy. For each type of 3D descriptor, the predictions achieved an R^2^ of at least 0.93 and a MAE of less than 0.04. We believe that this impressive capacity is attributable to the utilization of a conformation for each NP that has been optimized through alignment with the conformation obtained through molecular docking against the PD-L1 receptor (PDB ID 5N2F), as detailed in the Methods section (3.1. Datasets: Training and Test Sets). The most appropriate set of FPs and descriptors, comprising 1D&2D, along with all 3D descriptors, was selected for further investigation (see Table 6).

As with the QSDAR model, there is a decrease in the predictive capacity of the best model for the test set. However, in this case, the predictions are considered acceptable, with an R^2^ of 0.77 for the test set (see Table 6).

### 2.4. QSDAR Classification Model

As stated in Section 2.2.3, the predictive capacity of the most accomplished QSDAR regression model for the test set was determined to be inadequate. Consequently, a comparison was made of the predictive capacity of the QSDAR models for classification. The construction of the QSDAR classification model involved the selection of the 200 most relevant descriptors (NMR SPINUS spectral descriptors) and the implementation of LightGBM as the ML technique, as outlined in Section 2.2.3. It is further noted that the training and test sets were utilized in a manner consistent with the previously described approach (Section 2.2); however, with this approach, the calculated pIC_50_ values were substituted with the activity classes established for PD-L1 in Section 2.2.1. The training and test sets comprise 119,733 and 1202 NPs, of which 46,950 and 491 are active, and 72,783 and 711 are inactive, respectively. The LightGBM ML technique was employed to construct a QSDAR classification model for predicting PD-1/PD-L1 inhibition. The performance of this model was successfully evaluated through internal validation (ten-fold cross-validation for the training set) and external validation (the test set), as illustrated in Table 7.

**Figure 4:**
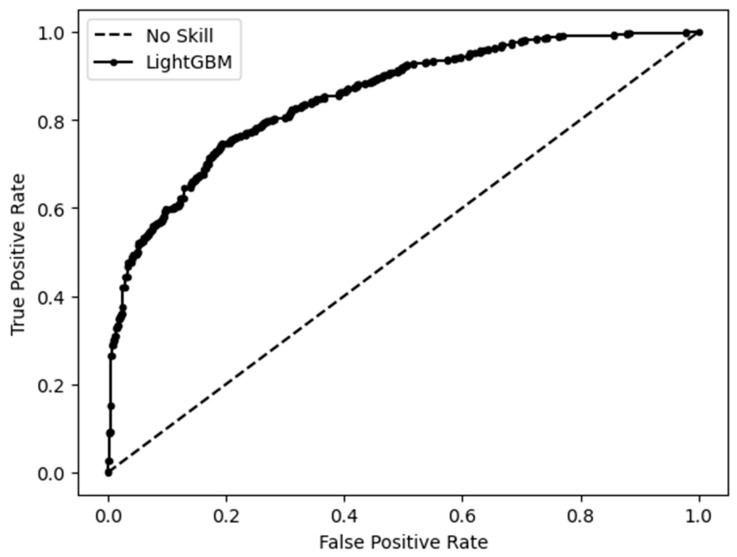
ROC obtained for the test set with the QSDAR classification model.

Unlike the results observed with the QSDAR regression model, the QSDAR classification model showed slightly better predictive performance on the test set than on the training set, based on a ten-fold cross-validation estimation. The model achieved an overall predictive accuracy (Q) of 0.78 and a Matthews correlation coefficient (MCC) of 0.55 for the test set, compared to 0.75 and 0.49, respectively, for the training set. The receiver operating characteristic curve (ROC) obtained for the test set with the LightGBM model trained with the 200 most important descriptors (NMR SPINUS spectral descriptors) is displayed in Figure 4. The QSDAR classification model, implemented with the LightGBM algorithm, achieved an area under the ROC curve (AUC) of 0.851.

#### Analysis of Outliers

The QSDAR classification model, trained with the 200 most relevant descriptors (NMR SPINUS spectral descriptors), predicted the test set with an accuracy of 0.78 and a MCC of 0.55. The ROC curve in Figure 4 illustrates the significance of the probabilities assigned to the predictions by the LightGBM models. Among the 1202 predicted NPs, the 18 FPs and 23 FNs with a probability higher than 0.8 were manually inspected to find possible reasons for incorrect predictions with high assigned probabilities.

**Figure 5:**
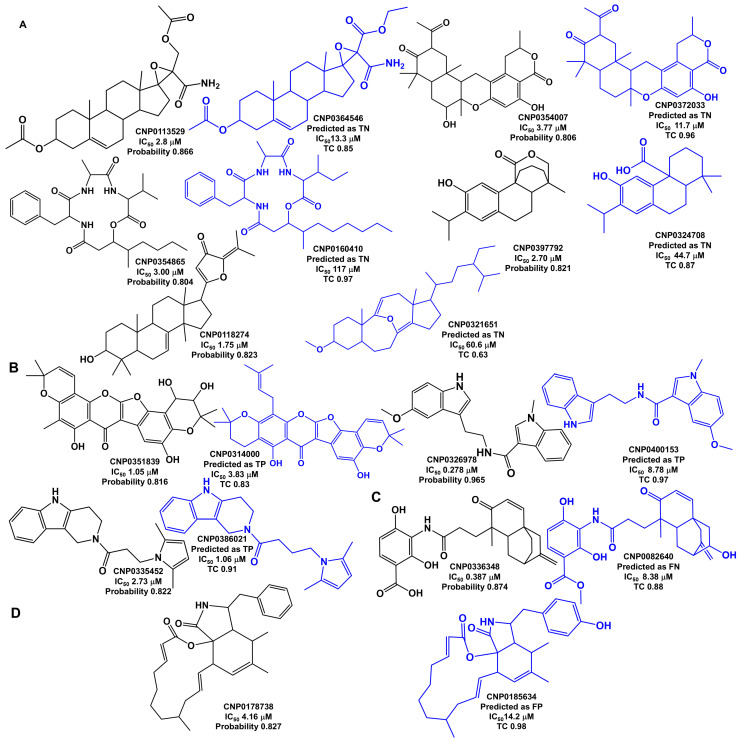
The chemical structures of ten FNs (CNP0113529, CNP0354007, CNP0354865, CNP0397792, CNP0118274, CNP0351839, CNP0326978, CNP0335452, CNP0336348, and CNP0178738) predicted with high probability, and their most similar training set counterpart structures (CNP0364546, CNP0372033, CNP0160410, CNP0324708, CNP0321651, CNP0314000, CNP0400153, CNP0386021, CNP0082640, and CNP0185634), highlighted in blue, are sorted as the training set predicted: (**A**) TN, (**B**) TP, (**C**) FN, and (**D**) FP. TC is the Tanimoto coefficient.

Most FNs (13 out of 23) are NPs with IC_50_ values calculated to be above 5 µM, placing them near the threshold for inhibitory activity, which is defined as an IC_50_ of 10 µM or lower. The remaining 10 FNs are NPs with IC_50_ values calculated to be less than or equal to 5 µM: four terpenoid derivatives (CNP0113529, CNP0354007, CNP0397792, and CNP0118274), two organoheterocyclic derivatives (CNP0326978 and CNP0335452), two polyketide derivatives (CNP0351839 and CNP0178738), one cyclic petide derivative (CNP0354865), and one benzoic acid derivative (CNP0336348); Figure 5. Five (CNP0113529, CNP0354007, CNP0354865, CNP0397792, and CNP0118274) of these ten predictions could be explained by similar molecules in the training set, which were assigned to the inactive class based on the IC_50_ calculated data. The ten structures of FN in Figure 5 were subjected to a similarity search against the training set, using fingerprints and Tanimoto coefficients. NPs CNP0113529, CNP0354007, CNP0354865, CNP0397792, and CNP0118274 have a similar counterpart in the training set assigned to the inactive class and are predicted as inactive in the ten-fold cross-validation estimation: CNP0364546 (Tanimoto coefficient of 0.85), CNP0372033 (Tanimoto coefficient of 0.96), CNP0160410 (Tanimoto coefficient of 0.97), CNP0324708 (Tanimoto coefficient of 0.87), and CNP0321651 (Tanimoto coefficient of 0.63), respectively. Likewise, the NP CNP0336348 has a similar corresponding molecule in the training set predicted to be inactive, CNP0082640 (Tanimoto coefficient of 0.88). The remaining four FN predictions (CNP0351839, CNP0326978, CNP0335452, and CNP0178738) can no longer be explained by the similar molecules in the training set, as was the case for the other six FNs. NPs CNP0351839, CNP0326978, CNP0335452, and CNP0178738 have a similar counterpart in the training set that is predicted as active in the ten-fold cross-validation estimation: CNP0314000 (Tanimoto coefficient of 0.83), CNP0400153 (Tanimoto coefficient of 0.97), CNP0386021 (Tanimoto coefficient of 0.91), and CNP0185634 (Tanimoto coefficient of 0.98), respectively.

**Figure 6:**
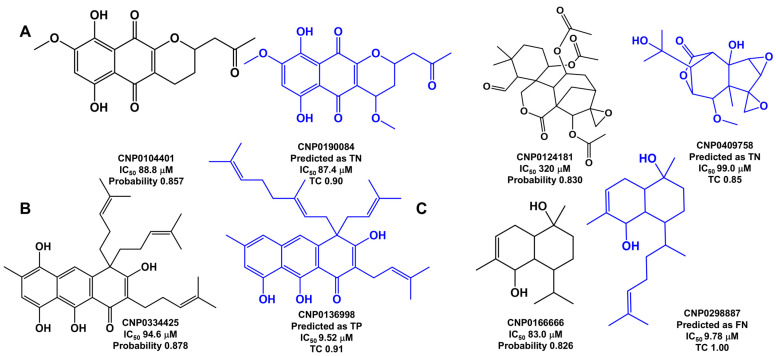
The chemical structures of four FPs (CNP0104401, CNP0124181, CNP0334425, and CNP0166666) predicted with high probability, and their most similar training set counterpart structures (CNP0190084, CNP0409758, CNP0136998, and CNP0298887), highlighted in blue, are sorted as the training set predicted: (**A**) TN, (**B**) TP, and (**C**) FN. TC is the Tanimoto coefficient.

Half of the FPs (9 out of 18) are NPs with calculated IC_50_ values below 20 µM, placing them near the threshold used to define inhibitory activity. The other nine FPs are NPs with IC_50_ values of more than 20 µM. Among these, four FPs have IC_50_ values of more than 80 µM: two terpenoid derivatives (CNP0124181, CNP0166666), one organoheterocyclic derivative (CNP0104401), and one polyketide derivative (CNP0334425); Figure 6.

As was the case with the six FNs, in the case of FP (CNP0334425), it has a similar molecule in the training set predicted with active CNP0136998 (Tanimoto coefficient of 0.91), as illustrated in Figure 6. In the remaining three FPs, as was the case with the other four FNs (see Figure 5), minor alterations in chemical structure may or may not have a significant impact on inhibitory activity. However, it appears that, in these NPs, the ML model was unable to grasp these effects.

### 2.5. Applicability Domain of PD-L1 QSDAR Classification Model

The applicability domain of a QSAR model highlights the part of a chemical space containing the compounds for which the model is able to provide reliable predictions. A Tanimoto coefficient (TC) matrix approach and the probabilities assigned to the predictions by the LightGBM classification model were used to define the applicability domain of the PD-L1 QSDAR model based on the similarity between a molecule in an external dataset, 1202 molecules in the test set, and all 119,733 molecules in the training set. The Tanimoto coefficient of similarity was calculated using an RDKit script [35]. A TC is a similarity value between 0 and 1 for a given pair of molecules. The closer the two molecular structures are to one another, the higher the TC value. By analyzing the TC matrix, the probabilities, and the class predictions obtained with the QSDAR classification model for the test set, we were able to define two thresholds based on the maximum TC value obtained by a given molecule in the test set and all the molecules in the training set and the probabilities assigned to the predictions. Accordingly, for a given molecule that the model has not previously encountered, the predicted values should be considered reliable if the molecule exhibits a maximum TC value greater than or equal to 0.90 in comparison to all the molecules of the training set or a probability greater than or equal to 0.8. When these thresholds are applied to the test set, 210 molecules are excluded. Considering the optimal QSDAR classification model, the SE and MCC for the 210 molecules outside the applicability domain of the model are 0.59 and 0.30, respectively, compared to the SE and MCC of 0.80 and 0.63 for the 992 molecules within the applicability domain. Available as Supplementary Information, File S1, are the SMILES strings of the 210 molecules outside the applicability domain of the model from the test set and the probabilities assigned to the predictions of the best QSDAR classification model.

### 2.6. Virtual Screening

As stated in the introduction, the two anticancer drugs, sonidegib and lapatinib, were identified as potential inhibitors of PD-L1 by our group by using an integrated CADD approach in a previous study [22]. Consequently, in order to validate the QSDAR classification model developed in the present study, it can be employed in the virtual screening procedure of the two anticancer drugs proposed in our previous study [22] and the positive control (BMS-200, Figure 2E). The most suitable model identified for the virtual screening procedure was the lightGBM classification model, which employed the 200 most significant NMR SPINUS spectral descriptors. The applicability domain of the QSDAR model was defined using two thresholds: a maximum TC value greater than or equal to 0.9 in comparison to all the molecules of the training set or a probability greater than or equal to 0.8. The application of these thresholds revealed that all three compounds present within the virtual screening library belonged to the applicability domain of the QSDAR classification model, exhibiting a probability greater than or equal to 0.8. The QSDAR model predicts that all three compounds are active against PD-L1. As mentioned in Section 2.1, the experimental IC_50_ value for BMS-200 (see Figure 2E) is 80 nM. This can be compared with the value of IC_50_ estimated by molecular docking, which was found to be 52.2 nM.

## 3. Materials and Methods

### 3.1. Datasets: Training and Test Sets

In April 2023, a total of 407,270 unique natural product (NP) entities were extracted from the COCONUT database (version from January 2022), https://coconut.naturalproducts.net (accessed on 3 April 2023) [7,8]. As discussed in Section 2.1 and illustrated in Figure 1, there is a correlation between the Gibbs free energy (ΔG_B_) calculated by molecular docking for the ligand-PD-L1 complex and the pIC_50_ for ligands with an average molecular weight of the molecule, ignoring hydrogens (HeavyAtomMolWt) < 500 Da. The SMILES strings of 172 molecules, experimental IC_50_, and calculated pIC_50_ by molecular docking are available as Supplementary Material, File S1. Consequently, the NPs with a HeavyAtomMolWt of less than 500 Da and the NPs that could be molecular docked were selected, yielding a total of 191,399 NPs. Subsequently, NPs for which the ΔG_B_ calculated by molecular docking had unusual values (greater than −2 kcal/mol and less than −20 kcal/mol) were also removed, resulting in a total dataset of 144,635 NPs. The RDKit and MolVS tools (https://molvs.readthedocs.io/en/latest/guide/intro.html, (accessed on 3 March 2025)) were utilized to standardize molecular structures by normalizing the tautomeric and mesomeric groups, aromatizing, and by removing small, disconnected fragments. Three-dimensional models of molecular structures were generated with the software program OpenBabel (version 2.3.1) [33]. ^1^H NMR SPINUS descriptors containing the discrete values of the ^1^H chemical shifts (285 spectral descriptors) and coupling constants (285 spectral descriptors) were calculated using the SPINUS program, https://neural.dq.fct.unl.pt/spinus/, (accessed on 3 March 2025) [33]. Empirical molecular fingerprints (FPs), 1D&2D molecular descriptors, and 3D molecular descriptors were calculated for the datasets using RDKit [35]. Various types of FPs with different sizes were calculated and explored, including 166 MACCSs (MACCS keys), 1024 CDKs (circular fingerprints), and 2048 RDKits (RDKit fingerprints) [35]. As discussed in more detail subsequently, molecular docking against the PD-L1 protein was conducted on all NPs from the complete dataset. The optimal docking conformation for each molecule, obtained by aligning the original prior-docking SDF files, calculated with OpenBabel, with the SDF files obtained as output from docking, was utilized to calculate the 3D molecular descriptors. A total of 242 1D&2D molecular descriptors were employed, encompassing electronic, topological, and constitutional descriptors, as well as three types of 3D molecular descriptors: Autocorr3D (80 descriptors), Getaway (271 descriptors), and Radial Distribution Function, RDF (210 descriptors). Five quantum descriptors (εHOMO, εLUMO, HOMO−LUMO gap, the dipole moment (DM) calculated using empirical point natural bond orbital (NBO) charges [38], and DFT-DM) were calculated by ML approaches that were developed in our group for molecular orbital energies [36] and DM [37]. In total, the final dataset consisted of 120,936 NPs.

The approximate 100:1 partition for training and test sets was carried out randomly, comprising 119,733 and 1202 NPs, respectively. The built QSDAR and QSAR models were developed and externally validated using the training and test sets, respectively. SMILES strings of the datasets, along with the corresponding calculated pIC_50_ by molecular docking and the predicted pIC_50_ by the ML approach, are available as Supplementary Material, File S1.

### 3.2. Molecular Docking

In this study, a total of 295,551 small NPs were docked to PD-L1, and the correlation between activity and binding energy against PD-L1 for each molecule was analyzed. The conversion of the SDF files to PDBQT files was facilitated by the software program OpenBabel (version 2.3.1). The AutoDock Vina program (version 1.2.3) [39,40] was then employed for docking to the PD-L1 receptor (PDB ID: 5N2F, https://www.rcsb.org/structure/5N2F, (accessed on 3 March 2025)). Prior to docking, water molecules and ligands were removed from 5N2F using the AutoDockTools (http://mgltools.scripps.edu/, (accessed on 3 March 2025)). The search space coordinates were centered at X: 32.759, Y: 12.47, and Z: 134.541, with dimensions of X: 20,000, Y: 20,000, and Z: 20,000. Ligand tethering of the PD-L1 receptor was performed by regulating the genetic algorithm (GA) parameters using 10 runs of the GA criteria. The resulting docking binding poses were then subjected to visualization through the utilization of the PyMOL Molecular Graphics System, Version 2.0 (Schrödinger, LLC and Warren DeLano, UCSF Chimera [41], and the Protein-Ligand Interaction Profiler (PLIP) web tool [42]. To ensure the reliability of the experimental results, a positive control test was conducted. This test involved the docking of the inhibitor (i.e., BMS-200) from the X-ray structure of the PD-L1/inhibitor complex and the same inhibitor with the 3D optimization approach (i.e., OpenBabel). The docking scores of the final set of 120,935 small NPs against the PD-L1 protein are presented in File S1 of the Supplementary Information.

### 3.3. ML Techniques

#### 3.3.1. Random Forest (RF)

A random forest (RF) [43,44] was implemented as an ensemble of unpruned regression trees, which are created using bootstrap samples of the training set. For each individual tree, the best split at each node is defined using a randomly selected subset of descriptors. Each individual tree is created using a different training and validation set. The final prediction for an object is yielded by a RF as the average of the predictions of the individual regression trees. The predictions obtained for objects not included in the training set are compared to the target values, and the deviations are averaged in the out-of-bag (OOB) error estimation. In the experiments presented here, RFs were used for the development of regression models to estimate the calculated pIC_50_. RFs were grown with the scikit-learn implementation [45] of the RandomForestRegressor [46]. The number of trees in the forest was set to 1000, the number of descriptors available for each node was optimized, and the other parameters were used with default values.

#### 3.3.2. Support Vector Machines (SVM)

Support vector machines (SVMs) [45,47] map multidimensional data into a hyperspace (a boundary or hyperplane) through a nonlinear transformation (kernel function). Subsequently, linear regression is applied in this space, with the boundary defined by examples of the training set—the support vectors. In this study, the focus was on exploring SVM models with the scikit-learn implementation [45] of the LIBSVM software, version 3.31 [48]. The epsilon SVM-regression type was selected, and the kernel function was set to the radial basis function with the default gamma parameter. The parameter C was optimized within the range of 1–1000 through 10-fold cross-validation with the training set.

#### 3.3.3. Deep Learning Multilayer Perceptron Networks (_d_MLP)

The implementation of feed-forward neural networks was undertaken utilizing the open-source software library Keras version 2.2.5 [49] based on the Tensor Flow numerical backend engine [50]. These widely utilized software tools, written in Python, facilitate the development and application of deep neural networks. However, the primary challenge in applying _d_MLP lies in the design of an adequate network architecture. Following a series of experiments, the final optimal hyperparameter settings were selected for the study under consideration based on 10-fold cross-validation experiments with the training set. These settings are listed in Table 8.

#### 3.3.4. Light Gradient-Boosting Machine (LightGBM)

LightGBM is a gradient-boosting framework based on decision trees that increases the efficiency of the model and reduces memory usage. It supports a number of different boosting algorithms, such as Gradient-Boosting Decision Tree (GDBT), Dropouts that meet Multiple Additive Regression Trees (DART), and LightGBM models, which were explored with the scikit-learn implementation of the LightGBM software, version 4.6.0.99 [51].

The GDBT–LightGBM-regression type was chosen, and the number of trees was set to 500, with the parameters “max_depth” (which sets a limit on the tree depth), “num_leaves” (which specifies the number of leaves in a tree), and the parameter “feature_fraction” (which specifies the fraction of the descriptors to be considered in each iteration) were optimized through 10-fold cross-validation with the training set.

The GDBT–LightGBM-classification type was chosen, and the number of trees was set to 1000, with the parameters “max_depth” (which sets a limit on the tree depth), “num_leaves” (which specifies the number of leaves in a tree), and the parameter “feature_fraction” (which specifies the fraction of descriptors to be considered in each iteration) were optimized through 10-fold cross-validation with the training set.

## 4. Conclusions

This study’s findings indicate promising results in predicting activity classes against PD-L1 from docking scores using ML models. Notably, 83% of the molecules in the test set were predicted with high accuracy (MCC = 0.63). On the other hand, outliers—molecules with a MCC of 0.30—accounted for only 17% of the test set. These results suggest that the proposed strategy is effective for pure compounds. However, further optimization is needed, particularly in simulating mixture spectra by combining the spectra of individual compounds.

## Figures and Tables

**Table 1:** PD-L1 activity classes, drug-likeness characteristics and NP-likeness scores for the training and test sets.

Sets	#	PD-L1 Activity		Drug-Likeness		NP-Likeness Score ^5^
		Active ^1^	Inactive ^2^	MW_heatom_^3^	MolLogP ^4^	
Training	119,733	46,950 (39%)	72,783 (61%)	112,555 (94%)	100,534 (84%)	52,640 (44%)
Test	1202	491 (41%)	711 (59%)	1141 (95%)	1004 (84%)	519 (43%)

^1^ IC_50_ calculated value less than or equal to 10 µM. ^2^ IC_50_ calculated value higher than 10 µM. ^3^ HeavyAtomMolWt between 160–480 Da according to the Ghose rule [31]. ^4^ LogP between −0.4–5.6 according to the Ghose rule [31]. ^5^ NPL score between −1–1 to increase the drug similarity [12].

**Table 2:** Evaluation of the predictive performance of the two spectral descriptors for modeling the PD-L1 activity using the RF algorithm for the training set in OOB estimation. The best model is highlighted in bold.

Spectral Descriptors	# ^1^	R^2^	MAE ^3^	RMSE ^4^
SPINUS	570	0.907 ^2^	0.229	0.311
GNN	250	0.784 ^2^	0.346	0.444
SPINUS + GNN	**820**	**0.907** ^2^	**0.228**	**0.304**

^1^ N.° descriptors. ^2^ Coefficient of determination squared. ^3^ Mean absolute error. ^4^ Root mean squared error.

**Table 3:** Spectral descriptor selection of NMR SPINUS set for the QSDAR RF model of the PD-L1 activity for the training set in OOB estimation. The best model is highlighted in bold.

Model	# ^1^	R^2^	MAE ^3^	RMSE ^4^
SPINUS	50	0.90086 ^2^	0.23427	0.31782
	100	0.90644 ^2^	0.22927	0.31147
	150	0.90685 ^2^	0.22903	0.31102
	**200**	**0.90777 ^2^**	**0.22856**	**0.31030**
	250	0.90685	0.22902	0.31101

^1^ N.° descriptors. ^2^ Coefficient of determination squared. ^3^ Mean absolute error. ^4^ Root mean squared error.

**Table 4:** Exploration of diverse ML algorithms using the 200 most important descriptors (NMR SPINUS spectral descriptors) for both the training set and test set. The ML technique exhibiting the most optimal performance is highlighted in bold.

Model	R^2^	MAE ^2^	RMSE ^3^
Training set			
RF ^4^	0.908 ^1^	0.229	0.310
LightGBM ^5^	**0.941 ^1^**	**0.156**	**0.223**
SVM ^5^	0.531 ^1^	0.423	0.595
CNN ^5^	0.502 ^1^	0.446	0.612
Test set			
RF ^4^	0.489 ^1^	0.464	0.626
LightGBM ^5^	0.525 ^1^	0.441	0.601
SVM ^5^	0.396 ^1^	0.516	0.680
CNN ^5^	0.377 ^1^	0.528	0.695

^1^ Coefficient of determination squared. ^2^ Mean absolute error. ^3^ Root mean squared error. ^4^ OOB estimation. ^5^ 10-fold cross-validation estimation.

**Table 5:** Evaluation of the predictive performance of FPs, 1D&2D, and 3D molecular descriptors for modeling the PD-L1 activity using the LightGBM algorithm for the training set in 10-fold cross-validation estimation. The best models are highlighted in bold.

Descriptors		# ^1^	R^2^	MAE ^3^	RMSE ^4^
**FPs**	MACCS	166	0.8213 ^2^	0.1879	0.2618
	Morgan	1024	0.91 ^2^	0.0987	0.1365
	RDKit	2048	0.92 ^2^	0.077	0.1248
**1D&2D**		**425**	**0.9384** ^2^	**0.017**	**0.0255**
**3D**	Autocorr3D	80	0.9429 ^2^	0.0373	0.0519
	Getaway	271	0.9384 ^2^	0.0132	0.019
	RDF	210	0.9384 ^2^	0.0202	0.0292
3D		561	**0.9384** ^2^	**0.0091**	**0.0132**
**Quantum**		5	0.6246 ^2^	0.333	0.4250

^1^ N.° descriptors or FPs. ^2^ Coefficient of determination squared. ^3^ Mean absolute error. ^4^ Root mean squared error.

**Table 6:** Prediction of the LightGBM ML algorithm with 1D&2D and 3D descriptors for both the training set and test set.

Model	# ^1^	R^2^	MAE ^3^	RMSE ^4^
	Training set ^5^			
1D&2D&3D	986	0.9384 ^2^	0.0071	0.0105
	Test set			
1D&2D&3D	986	0.7707 ^2^	0.2363	0.3112

^1^ N.° descriptors. ^2^ Coefficient of determination squared. ^3^ Mean absolute error. ^4^ Root mean squared error. ^5^ 10-fold cross-validation estimation.

**Table 7:** Prediction of the LightGBM ML algorithm with 200 of the most important descriptors (NMR SPINUS spectral descriptors) for both the training set and test set.

	Sets	
	Training ^1^	Test
TP ^2^	33,333	365
TN ^3^	57,268	574
FP ^4^	15,515	137
FN ^5^	13,617	126
SE ^6^	0.710	0.743
SP ^7^	0.787	0.807
Q ^8^	0.757	0.781
MCC ^9^	0.494	0.549

^1^ Ten-fold cross-validation estimation. ^2^ True positive. ^3^ True negative. ^4^ False positive. ^5^ False negative. ^6^ Sensitivity: the ratio of true positive to the sum of true positive and false positive. ^7^ Specificity: the ratio of true negative to the sum of true negative and false negative. ^8^ Overall predictive accuracy: the ratio of the sum of true positive and true negative to the sum of true positive, true negative, false positive, and false negative. ^9^ Matthews correlation coefficient.

**Table 8:** Hyperparameter settings of the best _d_MLP model.

Hyperparameter	Setting
Initializer	Glorot uniform
Number of hidden layers	4
Number of neurons in the 1st, 2nd, 3rd, and 4th layers	150
Activation 1st–3rd layers	Relu
Activation 4th layer	Relu
Batch size	128
Optimizer	Adam
Epochs	500

## Data Availability

All data generated or analyzed during this study are included in the article and Supplementary Materials.

## Supplementary Materials

The following supporting information can be downloaded at: https://www.mdpi.com/article/10.3390/md23060247/s1, File S1
